# Antibacterial Activity of Selected Essential Oils against Foodborne Pathogens and Their Application in Fresh Turkey Sausages

**DOI:** 10.3390/antibiotics12010182

**Published:** 2023-01-16

**Authors:** Branislav Šojić, Predrag Ikonić, Sunčica Kocić-Tanackov, Tatjana Peulić, Nemanja Teslić, Miloš Županjac, Ivana Lončarević, Zoran Zeković, Milica Popović, Stefan Vidaković, Branimir Pavlić

**Affiliations:** 1 Faculty of Technology Novi Sad, University of Novi Sad, Bulevar cara Lazara 1, 21000 Novi Sad, Serbia; 2 Institute of Food Technology in Novi Sad, University of Novi Sad, Bulevar cara Lazara 1, 21000 Novi Sad, Serbia

**Keywords:** biogenic amines, essential oils, fresh sausages, shelf-life, turkey meat

## Abstract

Essential oils (EOs) isolated from different plant materials, namely *Origanum majorana* L., *Satureja hortensis* L., and *Satureja montana* L. (OMEO, SHEO, and SMEO, respectively), were used in fresh turkey sausage processing. The chemical composition and in vitro antimicrobial potential of selected EOs and their mixture were determined. The minimum inhibitory concentration (MIC) and minimum bactericidal concentration (MBC) against foodborne pathogens (*Escherichia coli*, *Salmonella* Enteritidis, and *Listeria monocytogenes*) ranged in the interval of 0.44–7.1 µL/mL. Fresh turkey sausages were produced with EOs addition and marked as follows: TOMEO—0.150 µL/g OMEO; TSHEO—0.150 µL/g SHEO; TSMEO—0.150 µL/g SMEO; TEOM—0.050 µL/g OMEO, 0.050 µL/g SHEO and 0.050 µL/g SMEO, and control (C) (without EOs). Microbiological profile and biogenic amines content in fresh turkey sausages were recorded during storage. The selected EOs and their mixture efficiently reduced bacterial growth and biogenic amines formation and accumulation. The lowest *Enterobacteriaceae* count and total biogenic amine (BA) concentration were determined through treatment TSHEO. The results of this study show that selected EOs could be useful in fresh turkey sausage processing in order to improve safety and shelf-life.

## 1. Introduction

In the past two decades, poultry meat production has gradually increased worldwide [1]. According to FAO predictions, in 2030, poultry meat production will reach an amount of nearly 151 million tons [2]. The increase in demand for poultry meat among consumers is related to its low cost of production, absence of cultural and religious restrictions, and relatively high nutritive potential [1]. The demand for turkey meat is also constantly increasing, and presently this type of poultry meat accounts close to 30% of total poultry meat production [2]. Turkey meat is marked as an essential part of healthy nutrition due to its high level of vital minerals (iron, zinc, phosphorus, potassium, and magnesium), which helps to maintain the normal functioning of the human immune and nervous system [3,4]. Additionally, turkey meat is characterized by high levels of easily digestible proteins (≤28%) and relatively low levels of fat (≤5%) and cholesterol [4]. Turkey meat also possesses high-quality sensory attributes (color, flavor, and texture) [4,5]. Concerning high nutritive and sensory quality, turkey meat is widely used in processing different types of meat products, including fresh sausages [5].

Fresh sausages are widely consumed processed meat products worldwide [6]. According to Serbian legislation [7], these products are manufactured by grinding and mixing meat (pork, beef, or poultry) with different components: table salt, spice mixtures, carbohydrates, and water. Fresh sausages are manufactured without food preservatives (nitrites and nitrates) and thermal treatments. Hence, these products are usually characterized by relatively high numbers of microorganisms, and they undergo spoilage even during refrigerated storage, resulting in a relatively short shelf-life [6]. Along with the microbial growth, proteolysis occurs, leading to a generation of polypeptides, peptides, free amino acids, aldehydes, organic acids, etc. [8,9,10]. Following this process, biogenic amines (BA) are formed and accumulated due to the metabolic activity of present decarboxylating bacteria. BA are the anti-nutritional organic bases of low molecular weight, which are regularly found in a range of food products (meat and meat products, fish, dairy products, fermented vegetables, beer, wine, etc.), and might have a toxicological influence on human health. Their content in meat products is affected by several factors, including the quality of raw material (meat hygienic status and composition, pH, water activity, etc.), ingredients (food additives, starter cultures, etc.), manufacturing practices, processing operations and conditions, and storage conditions (time, temperature, packaging methods, etc.) [10,11,12]. Thus, the assessment of BA in different types of meat products is essential as an indicator of food hygiene and freshness, as well as from toxicological viewpoint [12,13,14,15].

One of the critical approaches to decrease microbiological growth and prolong the meat products shelf-life is the usage of food additives, including nitrites and nitrates. However, these food additives are considered unhealthy [16].

Therefore, scientists in the field of meat technology are oriented towards various natural substitutes to synthetic additives, concentrating on plant extracts, including essential oils (EOs) [17]. EOs are remarkable since they are noticeable as GRAS (generally recognized as safe) and enjoy wide acceptance by consumers [18]. Several authors have already determined the strong antimicrobial potential of different essential oils in the processing of fresh sausages [14,19,20,21,22,23]. Hence, this study aimed to assess the antimicrobial and antioxidative potential of selected Eos, namely *Origanum majorana* essential oil (OMEO), *Satureja hortensis* essential oil (SHEO), *Satureja montana* essential oil (SMEO) and their mixtures (EOM), against foodborne pathogens in vitro and on the quality and shelf-life of fresh turkey sausages.

## 2. Results and Discussion

### 2.1. Chemical Composition of Applied Essential Oils

The chemical profile of three EOs obtained from the *Lamiaceae* species is given in Table 1 and chromatograms are given in Figure S1 (Supplementary Materials). GC-MS analysis showed that EOs consist of several groups of components, i.e., monoterpene hydrocarbons, oxygenated monoterpenes, sesquiterpene hydrocarbons, and oxygenated sesquiterpenes. The EOs of all samples showed a predominant presence of oxygenated monoterpenes, which is clearly visible in terms of the contents of major compounds in each sample: terpinen-4-ol (OMEO), thymol (SHEO), and carvacrol (SMEO).

OMEO samples obtained from *O. majorana* showed the most diverse terpenoid profile among exanimated EOs. Two major compounds identified in this sample were γ-terpinene and terpinen-4-ol, with relative percentages of 14.33 and 27.69%, respectively. Results are in accordance with the literature data, since it has been reported that the content of these two compounds in OMEO were 9.5 and 32.1% [24]. According to Ghazal et al. [25], *trans*-sabinene hydrate (25.18%) and terpinen-4-ol (24.92%) were the most abundant compounds in the Hungarian variety of the *O. majorana* essential oil, while the content of γ-terpinene (6.48%) was lower compared to the OMEO obtained in this work. The OMEO samples also showed a very balanced content of less abundant compounds. It was observed that 18 compounds exhibited relative percentages from 1 to 10% (Table 1). This could be particularly interesting since these compounds could contribute to the synergistic effects and improve the bioactive potential of the sample.

On the other hand, fewer compounds were identified in SHEO and SMEO essential oils obtained from *S. hortensis* and *S. montana*. It was observed that both samples had a similar content of oxygenated monoterpene phenols (Table 1). In SHEO, thymol and carvacrol comprised 41.10 and 9.99% relative percentages, while carvacrol (53.58%) was the most abundant in SMEO, while the content of thymol was negligible. This was expected since both plant species are from the genus *Satureja*, and the same observation was reported in a systematic review that evaluated the chemical profile of all *Satureja* species [26]. The content of major compounds was followed by *p*-cymene and γ-terpinene, which were similar in SHEO (10.20 and 9.77%) and (12.12 and 14.39%). Minor compounds that were identified in both samples with a relative percentage higher than 1% were α-thujene, α-pinene, 1-octen-3-ol, β-myrcene, α-terpinene, borneol, and *trans*-caryophyllene.

### 2.2. Minimal Inhibitory Concentration and Minimal Bactericidal Concentration of the Selected Essential Oils

The antibacterial potential of selected EOs (OMEO, SHEO, SMEO, and EOM) was expressed by the microdilution method (Table 2). Minimal inhibitory concentration (MIC) and minimal bactericidal concentration (MBC) are determined for the most common pathogens in meat processing: *Escherichia coli*, *Salmonella* Enteritidis, and *Listeria monocytogenes*.

The MICs of EOs against all tested pathogen bacteria were low and ranged in the interval 0.44–3.55 µL/mL. There is no agreement on the acceptable inhibition level for plant extracts and EOs when compared with standards. However, Duarte et al. [27] recommended a classification of plant extracts based on MIC results (strong inhibitors: MIC up to 500 μg/mL; moderate inhibitors: MIC between 500 and 1500 μg/mL; weak inhibitors: MIC above 1500 μg/mL). According to this recommendation, it was observed that all selected EOs and their mixture could be marked as strong inhibitors against pathogenic bacteria: *E. coli, S.* Enteritidis, *and L. monocytogenes*. In the case of bactericidal potential, the MBC of SHEO was lower than those determined for OMEO and SMEO against all three pathogenic bacteria. The strong antimicrobial effect of SHEO is probably related to its chemical profile and high content of terpenoids: thymol—41.10%; carvacrol—9.99% [28]. Additionally, Burt [29] suggested that essential oil that contains a high percentage of these terpenoids possesses strong antimicrobial potential. These terpenoids stimulate the weakening of the lipid cell membrane, initiating the leakage of cellular contents and, finally, bacterial death [30]. Additionally, it should be noted that the lowest MIC/MBC against *E. coli* was determined in the mixture of EOs. This could be the result of the synergistic effect of bioactive terpenoids contained in EOs [30].

### 2.3. Physicochemical Characteristics of Fresh Turkey Sausages

The pH and water activity (a_w_) values of fresh turkey sausages ranged from 6.15 to 6.37 and from 0.949 to 0.960, respectively (Table 3). The pH of all the treatments decreased (*p* < 0.05) during the initial 24 h of storage. This could be attributed to the formation of organic acids, primarily lactic acid, due to the metabolic activity of lactic acid bacteria (LAB) [16]. Additionally, a significant (*p* < 0.05) increase in the pH values in all the treatments was recorded on 2nd day of storage. This was most likely related to the accumulation of alkaline compounds, including peptides, amines, and amino acids [31]. At the end of storage, the difference between treatments was approximately 0.05 pH units. Therefore, it was concluded that the addition of selected EOs did not affect (*p* > 0.05) the pH values of fresh turkey sausages.

**Figure 1:**
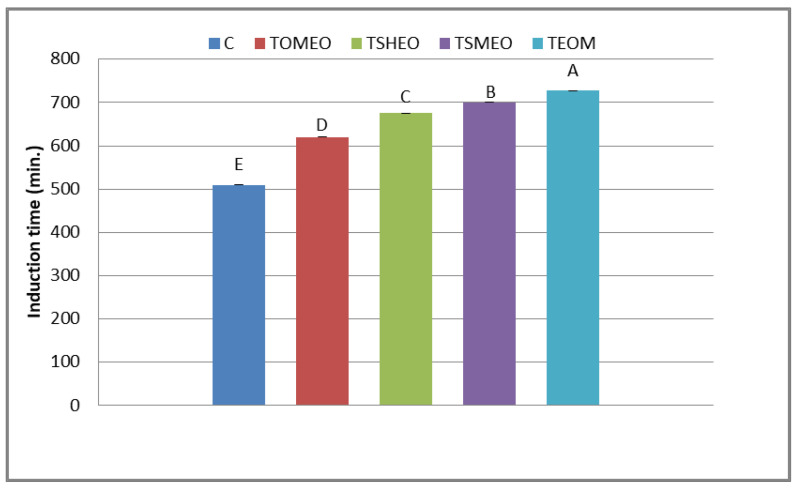
Oxidative stability of fresh turkey sausages. TOMEO—0.150 µL/g *O. majorana* essential oil; TSHEO—0.150 µL/g *S. hortensis* essential oil; TSMEO—0.150 µL/g *S. montana* essential oil; TEOM—0.050 µL/g *O. majorana* essential oil; 0.050 µL/g *S. hortensis* essential oil; and 0.050 µL/g *S. montana* essential oil. Control (C). Different upper cases in superscripts (A–E) indicate difference (*p* < 0.05) between treatments.

The antioxidant potential of fresh turkey sausages is displayed in Figure 1. The induction period is an interval (min) until oxygen pressure drops by 10% from the recorded maximum pressure. The registered values of this indicator ranged in the interval from 511 min (C) to 717 min (TEOM). The differences among the treatments were set as follows: TEOM ≥ TSMEO > TSHEO > TOMEO > C. Thus, the addition of selected EOs and their mixture slowed down the consumption of oxygen, i.e., provided the better oxidative stability of fresh turkey sausages. This finding could be the consequence of the antioxidant potential of terpenoids: terpinen-4-ol (OMEO), thymol (SHEO), and carvacrol (SMEO). The strong antioxidant potential of terpinen-4-ol was previously observed by Li et al. [32]. Moreover, de Oliveira et al. [33] suggested that thymol and carvacrol efficiently reduced lipid oxidation in meat products by scavenging free radicals. Due to the strong antioxidant capacity of these terpenoids, SMEO influenced the efficient reduction of lipid oxidation in fresh pork sausages during cold storage [34].

### 2.4. Microbiological Profile of Fresh Turkey Sausages

The microbiological profile of fresh turkey sausages is listed in Table 4. On the 1st day of storage, the total plate count (TPC) fluctuated from 4.33 log cfu/g (TEOM) to 5.44 log cfu/g (C). During the storage, TPC significantly (*p* < 0.05) increased for all five treatments. On 3rd day of storage, acceptable TPC was determined in TOMEO and TSHEO. According to the European Commission [35], the adequate microbiological quality of ground meat is reached when the TPC is lower than 6 log cfu/g. At the end of storage, the following differences in TPC were determined: C > TSHEO ≥ TSMEO > TEOM ≥ TOMEO. Generally, the addition of EOs significantly (*p* < 0.05) reduced TPC [29]. These outcomes established the strong antibacterial potential of selected EOs, particularly OMEO. The antimicrobial effect of *Origanum* species positively correlates with terpinen-4-ol content as the most dominant compound of OMEO [28]. Moreover, Cox et al. [36] observed that terpinene, as one of the main compounds of OMEO, inhibits the oxidative respiration of bacterial strains, causing damage to cytoplasmic membranes. According to this study, Yasar et al. [37] determined that OMEO possess a strong antimicrobial capacity regarding TPC in fresh beef meat.

*Enterobacteriaceae* count (TEC) and LAB counts generally increased during storage (Table 4). All three EOs and their mixture efficiently reduced TEC and LAB counts compared to C. The differences for both TEC and LAB counts were determined as follows: C > TSMEO > TOMEO > TEOM > TSHEO, at the end of storage. Generally, these bacteria possess very similar sensitivity against selected EOs. SHEO possesses the highest antimicrobial potential against the aforementioned microorganisms. This could be the consequence of the antimicrobial potential of thymol and carvacrol [29]. Carvacrol is marked as the terpenoid phenol with the highest antimicrobial potential. The high content of carvacrol causes the increased permeability of cell membranes. It simultaneously affects a reduction in pH gradient across the cytoplasm membrane, as well as the inhibition of ATP synthesis and, finally, the death of bacterial cells [28]. In this study, the highest antimicrobial potential against TEC and LAB was determined in the TSHEO. Although SHEO contains a lower percentage of carvacrol than SMEO, the most pronounced antimicrobial potential of SHEO could be the consequence of the synergistic effect of thymol and carvacrol with minor terpenoids, including *trans*-caryophyllene (3.25%) and *β*-bisabolene (3.45%). Carneiro et al. [38] and Ghavam et al. [39] determined the strong antibacterial potential of *β*-bisabolene and *trans*-caryophyllene.

The strong antimicrobial potential of SHEO in minced poultry meat was determined by Azimi et al. [40]. Moreover, SHEO efficiently reduced the growth of *Staphylococcus aureus* and *E. coli* in minced beef meat [41]. Previously, the antimicrobial potential of SMEO was confirmed, as it reduced the growth of TEC in fresh pork sausage when applied in similar concentrations (0.075–0.150 µL/g) [34].

Foodborne pathogens (*E. coli*, *L. monocytogenes* and S. spp.) were not detected in any treatment of fresh turkey sausages analyzed in this study. This finding could be an indicator of good hygienic practices applied during manufacturing in the meat processing pilot plant.

### 2.5. Biogenic Amines Contents in Fresh Turkey Sausages

**Figure 2:**
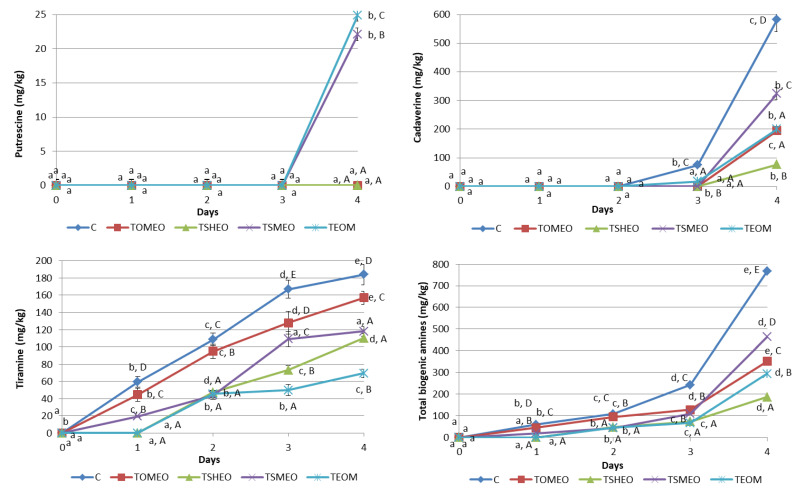
Evolution of biogenic amines (mg/kg) in fresh turkey sausages during storage. TOMEO—0.150 µL/g *O. majorana* essential oil; TSHEO—0.150 µL/g *S. hortensis* essential oil; TSMEO—0.150 µL/g *S. montana* essential oil; TEOM—0.050 µL/g *O. majorana* essential oil; 0.050 µL/g *S. hortensis* essential oil; and 0.050 µL/g *S. montana* essential oil; Control (C). Different upper cases (A–E) indicate difference (*p* < 0.05) between treatments at the same storage day. Different lower cases (a–e) indicate difference (*p* < 0.05) between different storage day, for each treatment.

Six BA were analyzed in samples of fresh turkey sausages using the HPLC method, and the determined BA profile is presented in Figure 2. Tryptamine (TRY), phenylethylamine (PHE), and histamine (HIS) were not detected in any sausage sample during four days of storage, while tyramine (TIR) was the dominant amine found in all samples starting from the first day until the end of storage, except in treatments TSHEO and TEOM after one day. Values of TIR ranged from 19.3 mg/kg (TSMEO, 1st day) to 184 mg/kg (C, 4th day). The storage time significantly influenced (*p* < 0.05) this BA content in all treatments, except in TEOM, where it remained almost the same between the second and third day (*p* > 0.05). Additionally, treatment significantly influenced (*p* < 0.05) TIR formation, i.e., addition of EOs significantly (*p* < 0.05) reduced TIR concentration during the whole storage period. TIR level was higher than 100 mg/kg in C samples after only two days, in TOMEO and TSMEO after 3 days, and in TSHEO after 4 days. Thus it exceeded the previously reported threshold value for potential poisoning due to TIR [11,14,22]. On the other hand, the concentration of this BA in TEOM remained below this level throughout the storage period. This finding is in accordance with previously published results by several authors [9,12,15] regarding the positive correlation between the LAB counts and the concentration of TIR in sausages.

Putrescine (PUT) was found just in sausages from TSMEO (22.1 mg/kg) and TEOM (24.9 mg/kg), after four days of refrigerated storage. Cadaverine (CAD) was determined for the first time after 3 days in C (75.2 mg/kg) and TEOM (17.5 mg/kg) sausages. The highest value of CAD was found at the end of the storage period in C sample, being 582 mg/kg. On the contrary, the sausages made with the addition of EOs were characterized by a much lower level of CAD at the end of the storage period, varying in a relatively wide range, from 77 mg/kg (TSHEO) to 324 mg/kg (TSMEO). Thus, most of the obtained values for this BA were significantly different (*p* < 0.05), except the ones registered for TOMEO and TEOM. High levels of PUT and CAD can contribute to food poisoning even though they do not have a direct negative health effect. However, it is known that these BA can potentiate the toxicity of HIS [13,14]. Considering the fact that HIS was not registered in all tested sausages during the storage period, it can be concluded that the risk level of poisoning due to their consumption is very low. Nevertheless, the accumulation of PUT and CAD in meat products could be correlated with the growth and metabolic activity of contaminant bacteria, i.e., *Enterobacteriaceae*, indicating lower quality (freshness) of raw materials and processing hygiene [11,14,15].

Regarding the total BA concentration, the following order was registered: C > TSMEO > TOMEO > TEOM > TSHEO. It can be seen that the levels of total BA were in complete accordance with the previously shown results concerning TEC and LAB counts (Table 4), confirming these microorganisms’ significant influence on BA generation. The storage time had a significant influence (*p* < 0.05) on total BA content both in C and treated sausages (TOMEO, TSHEO, TSMEO, and TEOM). Additionally, the influence of added EOs on total BA concentration was significant (*p* < 0.05), being especially noticeable after 4 days of storage. According to the results obtained in this study, it could be concluded that adding selected EOs into basic sausage formulation significantly reduced (*p* < 0.05) the formation and accumulation of BA in fresh turkey sausage. This finding is consistent with the previously published results obtained by a number of authors investigating the effects of different plant EOs on BA levels in meat products [14,22,31].

## 3. Materials and Methods

### 3.1. Plant Material and Essential Oils

*O. majorana* L. (OM), grown in Serbia, was produced by the Geneza d.o.o., Kanjiža, Serbia. OMEO was obtained according to the official Ph. Eur. VII procedure [42]. *S. hortensis* L. (SH) and *S. montana* L. (SM) were produced by Herba d.o.o (Serbia). The procedure was repeated in order to collect the proper level of obtained EOs (OMEO, SHEO, and SMEO) for further application in fresh turkey sausage processing.

### 3.2. GC-MS Analysis of Essential Oils

The terpenoids profile of EOs was determined by GC system (7890A, Agilent Technologies, Santa Clara, CA, USA) coupled with MS detector (5975C, Agilent Technologies, USA) and capillary column (30 m × 0.25 mm, 0.25 μm; 19091S-433UI HP-5MSUI, Agilent Technologies, USA). The samples were dissolved and properly diluted in methylene chloride prior to GC analysis. Injection volume was set at 1 µL and prepared samples were injected into the GC system via autosampler (7683B, Agilent Technologies, USA). The mobile phase was helium (>99.9997%), which was set at a constant flow rate of 2 mL/min. The temperature regime of GC oven was set as follows: starting temperature was 60 °C; 0–30 min temperature was increasing up to 150 °C at a rate of 3 °C/min; 30–35 min temperature was increasing up to 250 °C at a of rate 20 °C/min; and 35–40 min temperature was constant at 250 °C for 5 min. The injection temperature was set at a 250 °C. The terpenoids were identified using the NIST database of MS spectra and the database in the literature [43]. For the additional confirmation of terpenoids, the linear retention indices (LRI) were calculated for all identified compounds and compared with LRI reported in the literature [43]. The final results were expressed as a relative percentage (%) ± standard deviation.

### 3.3. Antimicrobial Activity of Essential Oils

The antimicrobial activity of selected EOs and their mixture was performed using the microdilution method. The MIC and MBC of the EOs were determined using the broth microdilution method according to Kocić-Tanackov et al. [44]. The antimicrobial activity of selected EOs was evaluated on *Escherichia coli* ATCC 8739; *Salmonella enterica* subsp. *enterica* serovar Enteritidis (group D) ATCC 14028; and *Listeria monocytogenes,* ATCC 13932, obtained from the American Type Culture Collection.

The MIC was determined as the lowest EOs concentration that inhibited the growth in the well (clear broth suspension) but still showed slightly visible growth on the plate. MBC was determined at the EOs concentration that inhibited growth in the well and showed no visible growth on the plate (the presence of ≤2 cfu per plate is acceptable). All tests were performed in duplicates for each EO.

### 3.4. Preparation of Fresh Sausage

Fresh turkey sausages were manufactured in a meat processing pilot plant (Institute of Food Technology, Novi Sad, Serbia) according to principles of good manufacturing practice (GMP) and good hygienic practice (GHP). Firstly, fresh turkey shoulders were deboned and trimmed to remove visible fat and connective tissues. The prepared meat at 3 ± 1 °C was minced using a stainless steel electric meat grinder (Titan 70 mm, Slovenia) until 8 mm meat particles were obtained. Further, ground meat was manually mixed with the seasonings for approximately 5 min. The basic formulation of fresh turkey sausages involved 98% lean turkey meat and 2% salt. Formerly, the following treatments (5) were processed with the supplementation of EOs: TOMEO—0.150 µL/g OMEO; TSHEO—0.150 µL/g SHEO; TSMEO—0.150 µL/g SMEO; TEOM—0.050 µL/g OMEO; 0.050 µL/g SHEO; 0.050 µL/g SMEO; and control (C) (without EOs). The sample sausages were stuffed in natural casings (pig small intestines; Ø ≈ 32 mm) using stainless steel piston filler SC-13 STAR (Talleres Ramon SL, Spain). The all sample sausages were processed under the same hygienic conditions, which implied low ambient temperature (10 °C) and the usage of sanitized accessories and equipment. Subsequently, sausages (approximately 0.1 kg each) were stored in the refrigerator at 3 ± 1 °C for 4 days. Samples were taken at different periods, after 0, 1, 2, 3, and 4 days of refrigerated storage, consisting of three randomly selected fresh turkey sausages from each treatment.

### 3.5. Physicochemical Analysis of Fresh Turkey Sausages

The oxidative stability of fresh turkey sausages was analyzed by RapidOxy 100 (Anton Paar, Blankenfelde-Mahlow, Germany). The method was described in detail by Jovanović et al. [45]. The samples were subjected to analysis immediately after the stuffing.

The pH was measured using a digital pH meter Testo 205 (Testo SE & Co. KGaA, Titisee-Neustadt, Germany), which was calibrated using standard buffers (pH = 4.00 ± 0.05 and pH = 7.00 ± 0.01 at 20 ± 2 °C) before measurements. Water activity (a_w_) was measured using a LabSwift-a_w_ measuring instrument (NovasinaAG, Lachen, Switzerland).

### 3.6. Microbiological Analysis of Fresh Turkey Sausages

The following microbiological analyses were performed: TPC—total plate count [46], TEC—*Enterobacteriaceae* count [47]; LAB—lactic acid bacteria count [48]; *E. coli* [49]; *L. monocytogenes* [50]; *Salmonella* spp. [51]; and sulfite-reducing clostridia count [52]. Results were expressed as a log cfu/g.

### 3.7. Biogenic Amines Determination in Fresh Turkey Sausages

BA were analyzed using high-performance liquid chromatography—HPLC (Agilent 1200 series, Agilent Technologies, Inc., Santa Clara, CA, USA). Tryptamine (TRY), phenylethylamine (PHE), putrescine (PUT), cadaverine (CAD), histamine (HIS), and tyramine (TIR) were determined as their dansyl derivatives, after homogenization (T18 Basic Ultra Turrax; IKA-Werke GmbH & Co. KG) and extraction with 0.4 M perchloric acid solution [15]. The single analysis lasted for 12 min, and the system was equilibrated for 6 min before the next analysis.

### 3.8. Statistical Analysis

Statistical analysis was conducted using STATISTICA 14.0 (TIBCO Software Inc., Palo Alto, CA, USA). All data were shown as mean values with their standard deviation indicated (mean value ± SD). Differences among treatments were assessed using Duncan’s post hoc test and were considered significant at *p* < 0.05.

## 4. Conclusions

The most dominant compounds in selected EOs were: terpinen-4-ol (27.69%)—OMEO; thymol (41.10%)—SHEO; and carvacrol (53.58%)—SMEO. The SHEO and the mixture of selected EOs possess the highest antimicrobial potential against pathogen bacteria (*E. coli*, *S.* Enteritidis, and *L. monocytogenes*) in vitro. The highest antioxidant potential in sausage samples was determined in the mixture of EOs. Moreover, all three EOs and their mixtures influenced the reduction of TPC, TEC, and LAB counts in sausage samples during refrigerated storage. The supplementation of selected EOs into sausage formulation affected a significant reduction (*p* < 0.05) in the formation and accumulation of BA in fresh turkey sausages. TRY, PHE, and HIS were not detected in any sausage sample during four days of storage, while TIR was the predominant amine found in almost all samples starting from the 1st day. The highest concentration of a particular BA was registered for CAD at the end of the storage period in the C sample. At the same time, the sausages made with the addition of EOs were characterized by much lower levels of CAD. These data strongly suggest that all selected EOs and their mixture could be used as natural antimicrobials in fresh turkey sausages. Thus, meat processors could be strongly encouraged to use these EOs as natural additives in meat processing.

## Figures and Tables

**Table 1:** Chemical profile of selected essential oils.

Name	RT [min]	OMEO		SHEO		SMEO	
		RP [%]	SD	RP [%]	SD	RP [%]	SD
α-Thujene	3.767	1.67 ^iA^	0.06	1.67 ^hA^	0.07	1.44 ^ghB^	0.01
α-Pinene	3.883	0.70 ^noC^	0.02	1.49 ^hiA^	0.08	1.16 ^iB^	0.03
Camphene	4.169	nd	n/a	1.06 ^jkA^	0.08	0.46 ^lB^	0.04
Sabinene	4.672	3.54 ^e^	0.04	nd	n/a	nd	n/a
β-Pinene	4.768	1.29 ^kA^	0.04	0.13 ^nB^	0.02	0.11 ^oB^	0.01
1-Octen-3-ol	4.815	nd	n/a	1.59 ^hiA^	0.61	1.36 ^ghA^	0.10
β-Myrcene	5.064	1.11 ^lA^	0.02	1.53 ^hiA^	0.98	1.79 ^fA^	0.10
α-Phellandrene	5.408	0.58 ^noA^	0.01	0.38 ^lmnB^	0.03	0.28 ^mnC^	0.01
ẟ-3-Carene	5.567	nd	n/a	0.12 ^n^	0.01	nd	n/a
α-Terpinene	5.79	9.57 ^cA^	0.14	2.89 ^efB^	0.08	2.24 ^eC^	0.03
*p*-Cymene	5.954	3.49 ^eC^	0.01	10.20 ^bB^	0.25	12.12 ^cA^	0.15
*o*-Cymene+Limonene	6.065	2.39 ^g^	0.01	nd	n/a	nd	n/a
Eucalyptol (1,8-Cineole)	6.15	nd	n/a	nd	n/a	0.78 ^jk^	0.02
*o*-Cymene+β-Phellandrene	6.15	nd	n/a	2.13 ^g^	0.00	nd	n/a
β-Phellandrene	6.139	1.39 ^jk^	0.13	nd	n/a	nd	n/a
γ-Terpinene	6.928	14.33 ^bA^	0.13	9.77 ^cB^	0.05	14.39 ^bA^	0.05
*cis*-Sabinene hydrate	7.203	2.08 ^hA^	0.01	0.41 ^lmnB^	0.01	nd	n/a
Terpinolene	7.855	3.05 ^fA^	0.01	0.26 ^mnB^	0.02	0.14 ^noC^	0.01
*trans*-Sabinene hydrate	8.188	4.40 ^d^	0.16	nd	n/a	nd	n/a
Linalool	8.31	3.09 ^fA^	0.17	0.77 ^klC^	0.05	1.33 ^hB^	0.05
*cis*-Menth-2-en-1-ol	8.983	2.05 ^h^	0.04	nd	n/a	nd	n/a
*trans*-Menth-2-en-1-ol	9.643	1.30 ^jk^	0.01	nd	n/a	nd	n/a
Borneol	10.582	nd	n/a	2.52 ^fA^	0.01	1.48 ^gB^	0.02
Terpinen-4-ol	10.984	27.69 ^aA^	0.46	1.43 ^hijB^	0.01	0.86 ^jkC^	0.02
α-Terpineol	11.572	4.41 ^dA^	0.02	0.40 ^lmnB^	0.01	0.21 ^mnoC^	0.03
*cis*-Piperitol	11.768	0.67 ^no^	0.01	nd	n/a	nd	n/a
Estragole	11.89	0.89 ^m^	0.07	nd	n/a	nd	n/a
*trans*-Piperitol	12.255	0.72 ^no^	0.11	nd	n/a	nd	n/a
Thymol methyl ether	13.293	nd	n/a	0.33 ^mn^	0.02	nd	n/a
Carvone	13.616	0.64 ^no^	0.01	nd	n/a	nd	n/a
Carvacrol methyl ether	13.648	nd	n/a	0.66 ^lm^	0.01	nd	n/a
Linalool acetate	14.162	1.28 ^k^	0.04	nd	n/a	nd	n/a
Terpinen-4-ol acetate	15.872	1.47 ^j^	0.02	nd	n/a	nd	n/a
Thymol	16.02	0.69 ^noB^	0.06	41.10 ^aA^	0.39	0.17 ^noC^	0.01
Carvacrol	16.359	0.55 ^oC^	0.05	9.99 ^bcB^	0.16	53.58 ^aA^	0.31
δ-Elemene	17.286	0.22 ^p^	0.03	nd	n/a	nd	n/a
Neryl acetate	18.604	0.09 ^p^	0.00	nd	n/a	nd	n/a
Thymol acetate	18.837	nd	n/a	1.24 ^ijA^	0.02	0.88 ^jB^	0.04
Geranyl acetate	19.388	0.11 ^p^	0.00	nd	n/a	nd	n/a
*trans*-Caryophyllene	20.479	2.20 ^hC^	0.04	3.25 ^deA^	0.03	2.91 ^dB^	0.05
β-Gurjunene	20.908	nd	n/a	nd	n/a	0.13 ^o^	0.01
Aromadendrene	21.289	0.17 ^pA^	0.00	0.14 ^nB^	0.00	nd	n/a
α-Humulene (α-Caryophyllene)	21.845	0.12 ^pA^	0.00	0.11 ^nB^	0.00	0.10 ^oC^	0.01
γ-Muurolene	22.867	nd	n/a	nd	n/a	0.17 ^no^	0.01
Germacrene D	22.989	nd	n/a	nd	n/a	0.45 ^l^	0.01
Viridiflorene (Ledene)	23.540	nd	n/a	0.18 ^n^	0.00	nd	n/a
Bicyclogermacrene	23.543	0.77 ^mn^	0.02	nd	n/a	nd	n/a
β-Bisabolene	24.175	nd	n/a	3.45 ^dA^	0.03	0.74 ^kB^	0.05
γ-Cadinene	24.366	nd	n/a	nd	n/a	0.18 ^mno^	0.03
δ-Cadinene	24.651	0.07 ^pC^	0.00	0.21 ^nB^	0.01	0.31 ^mA^	0.01
n.i.	25.536	nd	n/a	0.08 ^n^	0.00	nd	n/a
Spathulenol	26.685	0.58 ^no^	0.04	nd	n/a	nd	n/a
Caryophyllene oxide	26.833	0.61 ^noA^	0.06	0.19 ^nC^	0.01	0.24 ^mnoB^	0.01

RT—retention time [min]; RP—relative percentage [%]; SD—standard deviation; nd—not detected; n/a—not applicable; n.i.—not identified. Means ± SD with different letters ^(A–C)^ in the same row are significantly different (*p* < 0.05); values with different letters ^(a–p)^ in the same column are significantly different (*p* < 0.05).

**Table 2:** Minimal inhibitory concentration (MIC) and minimal bactericidal concentration (MBC) of the selected essential oils.

Essential Oils	Test Microorganism					
	*Escherichia Coli*		*Salmonella* Enteritidis		*Listeria Monocytogenes*	
	MIC (µL/mL)	MBC (µL/mL)	MIC (µL/mL)	MBC (µL/mL)	MIC (µL/mL)	MBC (µL/mL)
OMEO	0.89	1.78	3.55	7.1	1.78	7.1
SHEO	0.89	1.78	0.44	0.89	0.44	0.89
SMEO	3.55	7.1	1.78	3.55	0.89	1.78
EOM	0.44	0.89	0.44	0.89	0.44	1.78

*O. majorana* essential oil (OMEO); *S. hortensis* essential oil (SHEO); *S. montana* essential oil (SMEO); Essential oils mixture (EOM).

**Table 3:** pH and water activity (a_w_) values of fresh turkey sausages.

pH Values					
Storage Day	Treatments				
	C	TOMEO	TSHEO	TSMEO	TEOM
0	6.35 ± 0.03 ^aA^	6.37 ± 0.03 ^aA^	6.37 ± 0.03 ^aA^	6.36 ± 0.03 ^aA^	6.37 ± 0.03 ^aA^
1	6.15 ± 0.02 ^dA^	6.17 ± 0.04 ^dA^	6.17 ± 0.02 ^dA^	6.18 ± 0.00 ^cA^	6.17 ± 0.02 ^cA^
2	6.26 ± 0.02 ^bA^	6.28 ± 0.01 ^bcA^	6.33 ± 0.02 ^bB^	6.35 ± 0.02 ^aBC^	6.36 ± 0.01 ^aC^
3	6.32 ± 0.01 ^aA^	6.31 ± 0.02 ^bA^	6.37 ± 0.02 ^aC^	6.34 ± 0.01 ^aA^	6.28 ± 0.02 ^bB^
4	6.22 ± 0.04 ^cA^	6.26 ± 0.04 ^cA^	6.24 ± 0.06 ^cA^	6.27 ± 0.05 ^bA^	6.24 ± 0.01 ^bA^
a_w_ Values					
Storage Day	Treatments				
	C	TOMEO	TSHEO	TSMEO	TEOM
0	0.960 ± 0.010 ^cB^	0.951 ± 0.002 ^cA^	0.954 ± 0.001 ^cAB^	0.958 ± 0.001 ^cAB^	0.951 ± 0.002 ^cA^
1	0.971 ± 0.002 ^dC^	0.959 ± 0.002 ^dA^	0.959 ± 0.002 ^dA^	0.954 ± 0.001 ^dB^	0.958 ± 0.001 ^dA^
2	0.958 ± 0.003 ^bcA^	0.956 ± 0.001 ^bcA^	0.951 ± 0.001 ^bcB^	0.952 ± 0.000 ^bcB^	0.956 ± 0.001 ^bcA^
3	0.948 ± 0.002 ^aA^	0.949 ± 0.001 ^aA^	0.956 ± 0.001 ^aC^	0.959 ± 0.002 ^aB^	0.959 ± 0.001 ^aB^
4	0.949 ± 0.002 ^abA^	0.950 ± 0.001 ^abAB^	0.951 ± 0.001 ^abAB^	0.954 ± 0.001 ^abC^	0.952 ± 0.001 ^abB^

TOMEO—0.150 µL/g *O. majorana* essential oil; TSHEO—0.150 µL/g *S. hortensis* essential oil; TSMEO—0.150 µL/g *S. montana* essential oil; TEOM—0.050 µL/g *O. majorana* essential oil; 0.050 µL/g *S. hortensis* essential oil; and 0.050 µL/g *S. montana* essential oil. Control (C). Means ± SD with different letters ^(A–E)^ in the same row are significantly different (*p* < 0.05); values with different letters ^(a–d)^ in the same column are significantly different (*p* < 0.05).

**Table 4:** Microbiological profile of fresh turkey sausages.

Total Plate Count—TPC (log cfu/g)					
Storage Day	Treatments				
	C	TOMEO	TSHEO	TSMEO	TEOM
1	5.44 ± 0.16 ^aD^	4.87 ± 0.02 ^aA^	4.67 ± 0.03 ^aC^	4.95 ± 0.01 ^aA^	4.33 ± 0.03 ^aB^
2	6.08 ± 0.07 ^bB^	5.76 ± 0.02 ^bA^	4.98 ± 0.02 ^bC^	6.07 ± 0.11 ^bB^	5.67 ± 0.03 ^bA^
3	6.50 ± 0.03 ^cC^	5.99 ± 0.01 ^cA^	6.00 ± 0.11 ^cA^	6.19 ± 0.02 ^cB^	6.09 ± 0.08 c^AB^
4	7.00 ± 0.05 ^dC^	6.12 ± 0.06 ^dA^	6.28 ± 0.00 ^dB^	6.26 ± 0.05 ^dB^	6.14 ± 0.06 ^dA^
Total *Enterobacteriaceae* Count—TEC (log cfu/g)					
Storage Day	Treatments				
	C	TOMEO	TSHEO	TSMEO	TEOM
1	2.66 ± 0.18 ^aC^	2.04 ± 0.04 ^aAB^	2.12 ± 0.12 ^aAB^	2.22 ± 0.06 ^aB^	1.96 ± 0.00 ^aA^
2	3.50 ± 0.02 ^bD^	3.02 ± 0.02 ^bC^	2.00 ± 0.00 ^bB^	2.80 ± 0.10 ^bA^	2.85 ± 0.05 ^bA^
3	3.93 ± 0.03 ^cE^	3.74 ± 0.04 ^cD^	3.39 ± 0.09 ^cC^	3.00 ± 0.00 ^cA^	3.15 ± 0.15 ^cB^
4	4.50 ± 0.02 ^dE^	3.52 ± 0.01 ^dC^	3.06 ± 0.02 ^dA^	4.09 ± 0.01 ^dD^	3.31 ± 0.01 ^dB^
Lactic acid Bacteria—LAB Count (log cfu/g)					
Storage Day	Treatments				
	C	TOMEO	TSHEO	TSMEO	TEOM
1	2.98 ± 0.02 ^aD^	2.50 ± 0.03 ^aA^	2.42 ± 0.12 ^aA^	2.79 ± 0.09 ^aC^	1.85 ± 0.00 ^aB^
2	3.61 ± 0.00 ^bD^	2.69 ± 0.09 ^bA^	2.15 ± 0.15 ^B^	2.93 ± 0.03 ^C^	2.74 ± 0.04 ^A^
3	3.26 ± 0.26 ^cC^	2.97 ± 0.07 ^cAB^	2.78 ± 0.0 ^cA^	3.65 ± 0.12 ^cD^	3.20 ± 0.05 ^cBC^
4	3.97 ± 0.07 ^dD^	3.06 ± 0.06 ^dA^	2.71 ± 0.11 ^dB^	3.40 ± 0.10 ^dC^	2.95 ± 0.17 ^dA^

TOMEO—0.150 µL/g *O. majorana* essential oil; TSHEO—0.150 µL/g *S. hortensis* essential oil; TSMEO—0.150 µL/g *S. montana* essential oil; TEOM—0.050 µL/g *O. majorana* essential oil, 0.050 µL/g *S. hortensis* essential oil; and 0.050 µL/g *S. montana* essential oil. Control (C). Means ± SD with different letters ^(A–E)^ in the same row are significantly different (*p* < 0.05); values with different letters ^(a–d)^ in the same column are significantly different (*p* < 0.05).

## Data Availability

The data presented in this study are available in article and Supplementary Material.

## Supplementary Materials

The following supporting information can be downloaded at: https://www.mdpi.com/article/10.3390/antibiotics12010182/s1, Figure S1: GC-MS Total ion chromatograms of (a) *Origanum majorana*; (b) *Satureja hortensis*; (c) *Satureja montana* essential oils.
