# Supplementary material for: Antibacterial Activity of Selected Essential Oils against Foodborne Pathogens and Their Application in Fresh Turkey Sausages
Source: Antibiotics (Basel). 2023 Jan 16;12(1):182. doi: 10.3390/antibiotics12010182 (PMC9855142; doi:10.3390/antibiotics12010182)
Supplement: Supplementary file 1 [file antibiotics-12-00182-s001.zip › antibiotics-2155635-supplementary.pdf]

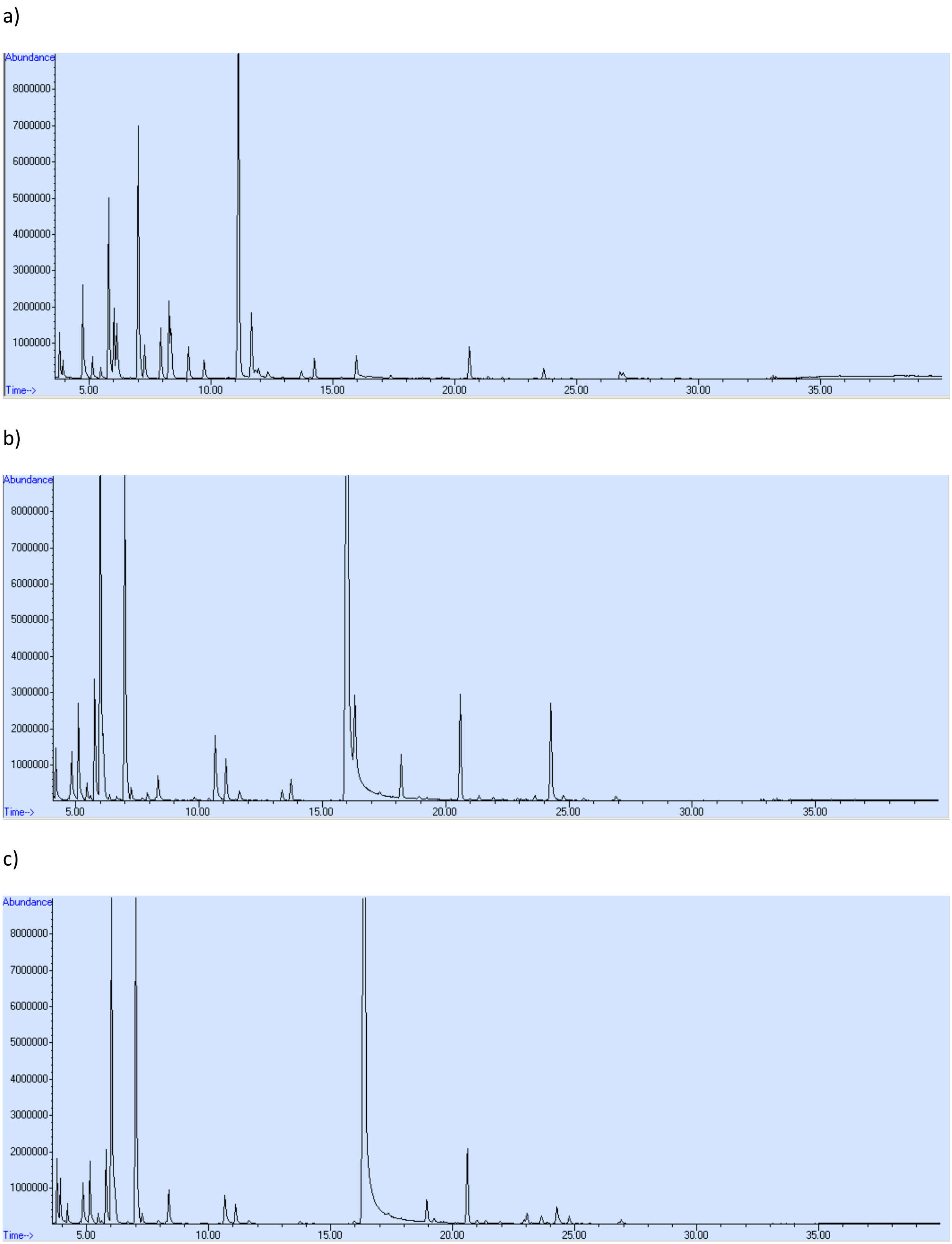

Figure S1. GC-MS Total ion chromatograms of a) *Origanum majorana*; b) *Satureja hortensis* c) *Satureja montana* essential oils
